# Study factors associated with the incompletion of clinical trials that include pediatric patients: a retrospective analysis of the European Clinical Trials Database and a lesson from the European region

**DOI:** 10.1186/s13063-021-05143-6

**Published:** 2021-03-12

**Authors:** Nanae Tanemura, Tsuyoshi Sasaki, Junko Sato, Hisashi Urushihara

**Affiliations:** 1 National Institute of Health and Nutrition, National Institutes of Biomedical Innovation, Health and Nutrition, Tokyo, Japan; 2 Chiba University Hospital, Chiba, Japan; 3 Pharmaceuticals and Medical Devices Agency, Tokyo, Japan; 4 Keio University, Tokyo, Japan

**Keywords:** Drug development, Pediatric clinical trial, Pediatric investigation plan, Study design, Study administration

## Abstract

**Background:**

Incomplete clinical trials for pediatric drug development result in a lack of adequate scientific evidence for providing appropriate medication to pediatric populations; this is especially true for Japan. Thus, using the European Clinical Trials Database (EudraCT), this study aimed to identify the factors related to the study design and administration that lead to incompletion of clinical trials that included pediatric patients.

**Methods:**

We focused on clinical trials that included patients under the age of 18 registered in the database, named as the European Clinical Trials Database between January 1, 2014, and December 31, 2018. Two groups of trials were identified: “all cases completed” and “not all cases completed,” reflecting whether they were completed in all participating countries/regions or not. To identify the factors of the occurrence of “not all cases completed,” a logistic regression analysis was performed to calculate the odds ratios and 95% confidence intervals. In total, 142 clinical trials (95 “all cases completed” and 47 “not all cases completed”) were analyzed.

**Results:**

The logistic regression analysis showed the number of countries in which a clinical trial was conducted to be the only significant factor (odds ratio: 1.3; 95% confidence interval: 1.1-1.5); this was identified as the primary factor for the occurrence of “not all cases completed” in the clinical trials that included pediatric patients.

**Conclusion:**

Our findings suggest that the feasibility of clinical trials that include pediatric patients, such as whether the countries in which the trial is to be conducted are suitable, must be considered prior to the trial.

**Supplementary Information:**

The online version contains supplementary material available at 10.1186/s13063-021-05143-6.

## Background

There are approximately 7000 known rare diseases, [1] 65% of which are serious and have no effective treatment. Two-thirds of these diseases can emerge by the age of 2 years, and 35% have a likelihood of causing death within 1 year [2].

In many countries, and especially in Japan, drug development for pediatric populations has not been adequately implemented, and this has been referred to as the “therapeutic orphan” dilemma [3]. Although some drugs contain labels describing the indications for administration or dosages for pediatric patients, off-label use of medications without official authorization for pediatric indications from regulatory agencies is prevalent. In Japan, while several off-label indications in pediatric patients are reimbursed by the public health insurance system in limited conditions [2], off-label use also frequently occurs in clinical practice within routine health care and its regulatory systems. The use of off-label or unlicensed drugs in Japan is entirely excluded from side effects therapy according to the Relief System for Sufferers from Adverse Drug Reactions by the Law for the Pharmaceuticals and Medical Devices Agency. This system denies any opportunity for recompense from the regulatory agency, even when side effects emerge in pediatric patients [2].

Several previous attempts to promote pediatric drug development in Japan have been made, including (1) investigator-initiated clinical trials, (2) the establishment of the Council on Unapproved Drugs/Off-Label Use to recommend the clinical development strategy for regulatory approval, (3) the extension of the statutory re-examination periods for drugs after their initial marketing authorization since 2010, and (4) additional premium pricing for pediatric drugs, which increased drug prices by 5–20% since 2016. However, the effectiveness of these countermeasures has been negligible since they have not been legally enforced in Japan.

In contrast, the USA has implemented regulations such as the Best Pharmaceuticals for Children Act in 2002 and the Pediatric Research Equity Act (PREA) in 2003, [4, 5] while the EU implemented the Pediatric Regulation in 2017 [5, 6]. Notably, pediatric clinical trials in the EU commence after phase 1, at an earlier stage than in the United States, to ensure that the drugs are simultaneously commercially available for all patients, including pediatric patients. However, even though such effective and efficient regulations for pediatric drug development exist, a limited number of clinical trials have been conducted, especially trials involving premature infants, infants, toddlers, and children receiving life support [7].

In contrast to the well-established clinical trial designs for adult patients, the availability of existing methodologies for pediatric clinical trials for the development of new drugs is relatively limited to the industry, academia, and regulators. Several pediatric clinical trials have proven to be less than successful, [8] further highlighting the need to examine clinical trial designs for pediatric drug development [9].

Thus, the aim of this study was to identify the factors related to the study design or study administration that was associated with incomplete clinical trials that included pediatric patients, using the European Clinical Trials Database (EudraCT).

## Methods

### Definitions for investigated clinical trials

In this study, the term “all cases completed” is used to define clinical trials that were conducted in single or multiple countries that achieved the status of “completed” in all geographic regions. Specifically, this term was used for the trials that have “been completed in accordance with the full requirements of the protocol” [10].

However, the “not all cases completed” label refers to clinical trials that were conducted in single or multiple countries that did not achieve the status of “completed” in all regions. Such clinical trials were additionally classified as either “not authorized,” “temporarily halted,” or “prematurely ended,” according to the definitions provided in the “How to search the EU Clinical Trials Register,” published by the European Medicines Agency (EMA) on April 28, 2014. “Not authorized” refers to “a trial for which a negative ethics committee opinion was issued” in any of EU member states, [10] and that could therefore not be initiated in that state. The label “temporarily halted” refers to “a trial that has been temporarily interrupted. Reasons for such an interruption are varied, ranging from an interruption in the supply of an investigational medicinal product that needed to wait for the authorization of a substantial amendment to the protocol.” “Prematurely ended” refers to “a trial that ended before the completion of the all procedures described in the protocol. Reasons for a premature end can be related to the lack of product safety or efficacy, or lack of trial feasibility.”

### Data source

To obtain the data of interventional clinical trials, we systematically searched EudraCT database [11]. EudraCT is a European database developed by the EMA for all interventional clinical trials on medicinal products authorized in the European Union (EU) and those authorized outside the EU/European Economic Area (EEA) provided they formed part of a Pediatric Investigation Plan (PIP) from 1 May 2004 onwards. This database contains clinical trial protocols and study results that are publicly and readily available without any limitations of use (https://www.clinicaltrialsregister.eu/ctr-search/search). All clinical trials in this database are coded with a EudraCT number.

### Trial selection

The trial data were collected from EudraCT using the advanced search engine in this site. The eligible trials were defined according to the following criteria: (1) all countries “except for those outside EU/EEA,” (2) the date (between January 1, 2014, and December 31, 2018) when the trial information was first entered into the EudraCT database by a competent national authority or a third country data provider, and (3) the study status of “trials with results.” These searches were conducted on February 7, 2019. The target clinical trials comprised interventional phase 2 or 3 trials including pediatric subjects (newborns: 0–27 days; infants and toddlers: 28 days–23 months; children: 2–11 years; and adolescents: 12–17 years).

Next, we classified the trials based on the above definitions for investigated clinical trials. The clinical trials that were not initiated at all did not meet the definitions for “all cases completed” or “not all cases completed” and were therefore excluded from the analysis.

### Data collection and variables

We collected the data related to the study design of each clinical trial for analysis. For instance, we examined the type of disease on which the clinical trial focused, whether the disease was rare, the trial phase, whether the trial was randomized, whether the trial was double-blind, the number of enrolled participants, whether the trial included infants and toddlers, the number of countries in which the clinical trial was conducted, the year the clinical trial started, and the length of the trial period (in days).

Next, we collected data related to the administrative aspects of each clinical trial. We examined whether the clinical trial formed part of a pediatric investigation plan (PIP) authorized by a competent authority, the sponsor type, whether the clinical trial had undergone substantial protocol amendments, whether there were no protocol amendments after starting a clinical trial, whether the clinical trial was interrupted globally, and the trial’s “not all cases completed” status (i.e., “not authorized,” “temporarily halted,” or “prematurely ended”).

### Main outcome

The main outcome of this study was the identification of the factors that led to the occurrence of a “not all cases completed” status in clinical trials that included pediatric patients.

### Analysis

Data on the study design and administration of clinical trials were summarized by categories of “all cases completed” or “not all cases completed.” We calculated a median and a range (minimum–maximum) for continuous variables and a proportion for categorical variables. The differences between the categories “all cases completed” and “not all cases completed” were tested using chi-square tests or Fisher’s exact tests in the case of categorical variables, and the Mann-Whitney *U* test in the case of continuous variables. A two-sided alpha level of *p* <  0.05 was used for statistical significance.

To identify the factors influencing the occurrence of trials with “not all cases completed,” multivariate logistic regression analysis (simultaneous forced entry) was performed to calculate odds ratios (ORs) and 95% confidence intervals (95% CIs). The four variables were a priori fixed into the logistic model: PIP, rare disease, enrollment of “newborns” or “infants and toddlers,” and the number of countries in which the clinical trial was conducted. Spearman’s rank correlation coefficients were calculated to check for multicollinearity between every combination of two variables among the four forced-entry independent variables. All statistical analyses were performed using JMP® 14 (SAS Institute Inc., Cary, NC, USA).

### Ethics statement

Since this was a retrospective study using publicly available official materials published on the EMA websites, none of the data included personal information that would render the subjects identifiable. Accordingly, an ethical review was not required for this study.

## Results

### Trial selection

**Fig. 1:**
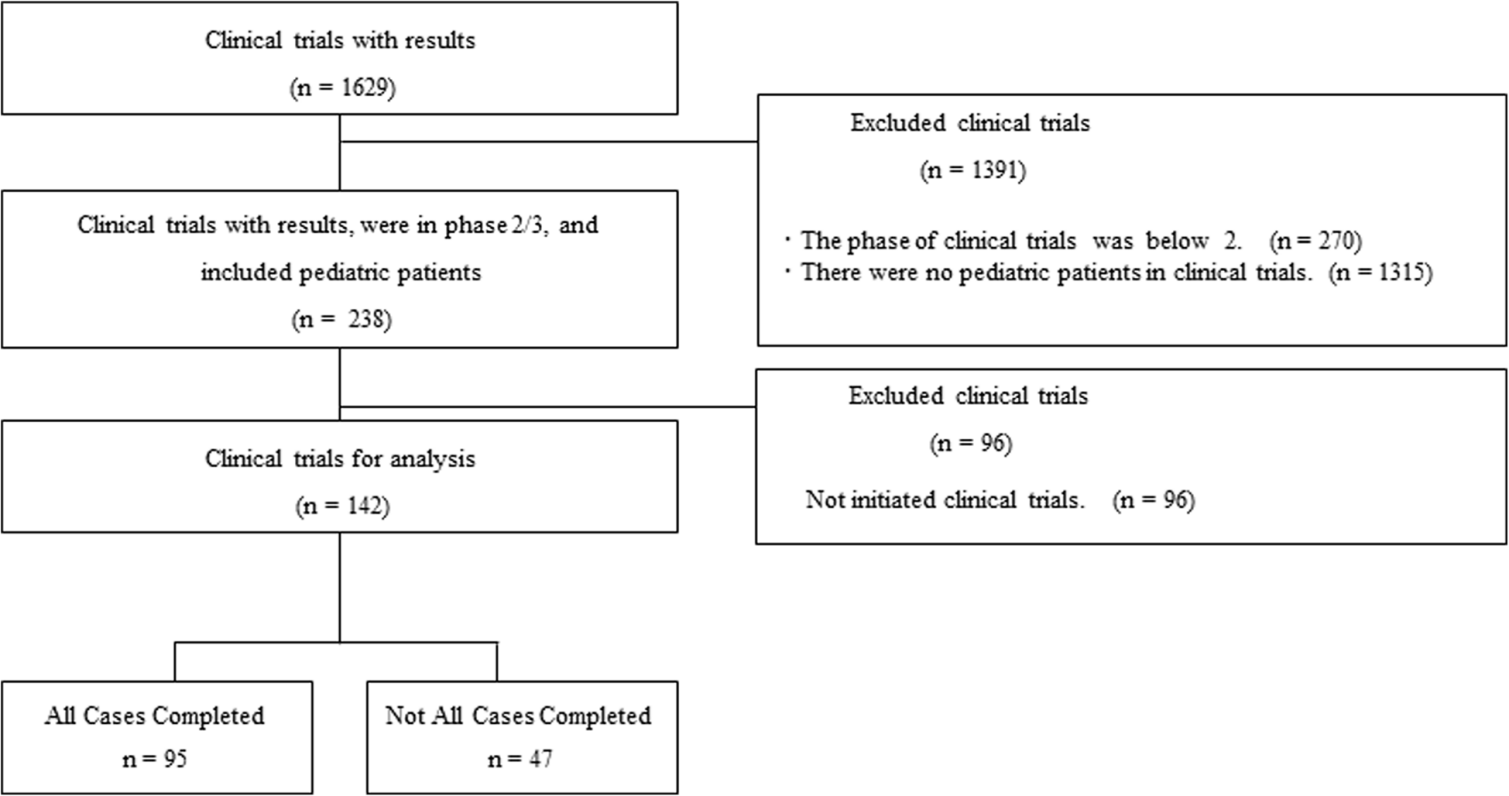
Flow diagram for study selection

At first, we extracted 1629 clinical trials with results from the EudraCT. From these, 1391 clinical trials were excluded. These included those in a study phase earlier than 2 (*n* = 270) and those excluding pediatric patients (*n* = 1315), leaving 238 clinical trials. Finally, 96 clinical trials that were not initiated due to not being authorized in any of the competent authorities in the EU were excluded (Fig. 1).

### Trial characteristics

In total, 142 clinical trials were identified and analyzed (95 “all cases completed” and 47 “not all cases completed”; Fig. 1). The study characteristics are summarized in Table 1. All of the clinical trials in the following disease categories were terminated without achieving the pre-specified number of study samples (that is, trials of the “not all cases completed” group): gastrointestinal disorders (two trials), pregnancy, puerperium and perinatal conditions (two trials), psychiatric disorders (two trials), reproductive system and breast disorders (four trials), and vascular disorders (two trials).

**Table 1:** Summary of the target diseases of the clinical trial

Items	Disease	All cases completed	Not all cases completed
		*n* = 95	*n* = 47
Disease: SOC Terms (multiple answers)	Blood and lymphatic system disorders	3	1
	Cardiac disorders	1	1
	Congenital, familial, and genetic disorders	22	7
	Ear and labyrinth disorders	0	0
	Endocrine disorders	1	1
	Eye disorders	2	1
	Gastrointestinal disorders	0	2
	General disorders and administration site conditions	0	0
	Hepatobiliary disorders	0	0
	Immune system disorders	0	0
	Infections and infestations	23	10
	Injury, poisoning, and procedural complications	0	0
	Investigations	3	0
	Metabolism and nutrition disorders	7	2
	Musculoskeletal and connective tissue disorders	4	0
	Neoplasms benign, malignant, and unspecified (incl. cysts and polyps)	2	2
	Nervous system disorders	9	1
	Pregnancy, puerperium, and perinatal conditions	0	2
	Product issues	0	0
	Psychiatric disorders	0	2
	Renal and urinary disorders	3	0
	Reproductive system and breast disorders	0	4
	Respiratory, thoracic, and mediastinal disorders	17	4
	Skin and subcutaneous tissue disorders	7	0
	Social circumstances	0	0
	Surgical and medical procedures	17	3
	Vascular disorders	0	2

### Design of clinical trials

For both groups, phase 3 was the most common trial phase (“all cases completed,” *n* = 50, 52.6%; “not all cases completed,” *n* = 22, 46.8%). Randomized clinical trials accounted for 58 of the “all cases completed” (61.1%) group and 30 of the “not all cases completed” (63.8%) group; however, double-blind trials accounted for less than 50% of the cases in both groups (“all cases completed,” *n* = 42, 44.2%; “not all cases completed,” *n* = 19, 40.4%). For both groups, the lowest median number of pediatric patients in the age categories of newborns and infants and toddlers was zero among the clinical trials. However, the “all cases completed” group had a slightly but significantly higher median number of adolescent participants (eight patients) compared with the “not all cases completed” group (one patient*; p* = 0.03). Further, the “not all cases completed” group had no children and one adolescent patient per trial on average. The proportion of clinical trials that included newborns or infants and toddlers was approximately 20% in both groups (“all cases completed,” *n* = 19, 20%; “not all cases completed,” *n* = 10, 21.7%). The median (minimum–maximum) number of the countries participating in the trials was two (1–10) for the “all cases completed” group, and four (1–18) for the “not all cases completed” group (*p* <  0.001). Finally, the median trial period was longer for the “not all cases completed” group, at 723.5 days, than in the “all cases completed” group, at 616 days (Table 2), but is insignificant.

**Table 2:** Summary of the study designs of the clinical trials

Items	Definition	All cases completed	Not all cases completed	*p* value
		*n* = 95	*n* = 47	
		*n* (%)	*n* (%)	
Rare disease	Yes	27 (28.4)	13 (27.7)	0.92
Phase	2	28 (29.5)	18 (38.3)	0.87
	2/3	2 (2.1)	0 (0)	
	3	50 (52.6)	22 (46.8)	
	Other	15 (15.8)	7 (14.9)	
Randomized	Yes	58 (61.1)	30 (63.8)	0.75
Double-blind	Yes	42 (44.2)	19 (40.4)	0.67
Clinical trials that included newborns or infants and toddlers	Yes	19 (20)	10 (21.7)	0.86
		Median, min–max	Median, min–max	
Number of enrolled participants per age categories	All age categories including over 18 years	111, 1–4176	42, 6–1177	0.02
	Newborn	0, 0–16	0, 0–4	0.43
	Infant and toddler	0, 0–1229	0, 0–110	0.67
	Child	2, 0–978	0, 0–506	0.07
	Adolescent	8, 0–448	1, 0–327	0.03
Number of countries in which the clinical trial was conducted		2, 1–10	4, 1–18	< 0.001
Year clinical trial started	2014	41 (43)	24 (51)	0.65
	2015	32 (34)	10 (21)	
	2016	16 (17)	10 (21)	
	2017	6 (6.3)	3 (6.4)	
Trial period (days)	Final date–start date + 1	616, 130–1570	723.5, 81–1524	0.18

*p* values; chi-square test for categorical variables/Mann-Whitney *U* test for continuous variables

### Study administration of clinical trials

In total, 18.9% (18 of 95) of the “all cases completed” group were planned by the PIP, compared to 27.7% (13 of 47) of the “not all cases completed” group *(p* = 0.24). The proportion of clinical trials with no protocol amendments after starting trials was approximately 40% for both groups. Global interruptions of the clinical trial were present in 14.9% of the “not all cases completed” group (*n* = 4, 8.5%), and the “prematurely ended” clinical status was detected in 43 of the cases in this group (91.5%; Table 3).

**Table 3:** Summary of the administration of the clinical trials

Items	Definition	All cases completed	Not all cases completed	*p* value
		*n* = 95	*n* = 47	
		*n* (%)	*n* (%)	
Clinical trial with an agreed PIP	Yes	18 (18.9)	13 (27.7)	0.24
Sponsor type	Commercial	87 (91.6)	47 (100.0)	0.10
Clinical trial with substantial protocol amendments	Yes	71 (74.7)	31 (66.6)	0.27
Clinical trial with no protocol amendments after starting trials	Yes	41 (43.2)	20 (42.6)	0.94
Global interruptions of the clinical trial	Yes	NA	7 (14.9)	NA
Clinical trial status				
Not authorized	Yes	NA	4 (8.5)	NA
Temporarily halted	Yes	NA	2 (4.3)	NA
Prematurely ended	Yes	NA	43 (91.5)	NA

*p* values; chi-square test for categorical variables

Note: *PIP* pediatric investigation plan, *NA* not applicable. Not authorized: Pediatric trials for which a negative ethics committee opinion was issued. Since a trial with a negative ethics committee opinion cannot proceed, these are labeled as not authorized. Temporarily halted: A trial that has been temporarily interrupted

Prematurely ended: A trial that has ended without completing all events described in the protocol. Reasons for a premature end can be related to lack of product safety or efficacy, or lack of feasibility of the trial

### Factors of the occurrence of “not all cases completed”

There was no multicollinearity suggested by the correlation analysis among these independent variables. The results of the logistic regression analysis for the identification of factors influencing the occurrence of “not all cases completed” are shown in Table 3. Only the number of countries in which the clinical trial was conducted was identified as a significant factor (OR: 1.3; 95% CI [1.1, 1.5]; Table 4).

**Table 4:** Factors related to incomplete clinical trials (*N* = 138)

Variable		Adjusted OR	95% CI
Trial part of an agreed PIP	Yes	1.8	[0.7, 4.3]
	Both	1.0	[0.1, 8.3]
	No	1.0	Reference
Rare disease	Yes	1.2	[0.5, 2.7]
	No	1.0	Reference
Clinical trials that included newborns or infants and toddlers	Yes	1.3	[0.5, 3.4]
	No	1.0	Reference
Number of countries in which the trial was conducted	Increment of one country	1.3	[1.1, 1.5]

Coefficient of determination; 0.09, Model goodness-of-fit; *p* = 0.007

Note: *PIP* pediatric investigation plan, *CI* confidence interval, *OR* odds ratio

## Discussion

This study sought to identify the factors related to the occurrence of “not all cases completed” in clinical trials by analyzing the key characteristics of clinical trials that included pediatric patients in the EudraCT database. The results showed that the number of countries in which a clinical trial was conducted was significant, which influenced the successful completion of clinical trials that included pediatric patients.

Most clinical trials that were included in the analysis were in phase 3 and conducted by commercial companies. A small number of clinical trials were planned by the PIP, targeted for a rare disease. There were no significant differences between the “all cases completed” and “not all cases completed” groups for these trial factors. We found that two variables significantly differed between the groups: the number of enrolled participants, particularly adolescents, and the number of countries where the clinical trials were conducted. The “not all cases completed” group had fewer patients enrolled than the “all cases completed” group, especially in the studies with adolescents, but involved a larger number of countries in the clinical trials. In a previous study that involved a systematic review of research conducted over the past 30 years regarding the reasons for failure of clinical trials, slow enrollment of patients was indicated as the reason why trials were expanded by adding new study sites or countries [12]. These changes may lead to ad hoc protocol amendments and delays in research progress. In another report, the main cause of study failure was reported to be a small sample size [13]. This finding suggests that a large number of participating countries could possibly be related to the premature termination of clinical trials, which may be ascribable to the potential burden of handling the different regulations and administrations of clinical trials across participating countries [13, 14]. Actually, lower numbers of patients were enrolled during the limited trial period in the “not all cases completed” group, which highlights problems reported for the multi-regional clinical trials, such as a lack of infrastructure and miscommunication. This may be the result of differences in the cultures and healthcare systems across the participating countries [15, 16].

### Future steps for pediatric clinical trials

Our findings suggest that, when conducting multi-regional clinical trials involving several countries, the feasibility of completing the trials as planned should be ensured. Several aspects should be considered in advance, including suitable trial design and effective administration, such as study procedures.

International cooperation for clinical trials that target small populations, such as rare disease or pediatric drug development, should be investigated further. Previous studies of EU-based trials have illustrated the importance of harmonization in clinical trial procedures where the trials conducted are necessary for pediatric clinical trials, given the rise of multicenter and multinational research [17]. It was reported that the key challenge these trials faced was overcoming the lack of harmonization of the status of several procedures across different countries. The International Council for Harmonization of Technical Requirements for Pharmaceuticals for Human Use (ICH) harmonized E17 guidelines, and the General Principles for Planning and Design of Multi-regional Clinical Trials adopted on 16 November 2017 [18] provide recommendations for study designs involving multi-regional clinical trials. Utilizing these guidelines might increase their acceptability in the global regulatory environment.

In addition, clinical trials involving small groups may require multi-regional or multi-site designs to efficiently address the issue of participant shortage. Additionally, increased efforts to enhance cooperation among study sites and regions are needed. Effective drug development planning has been described in Section 4 (“drug development planning”) and Section 4.4 (“feasibility”) of the ICH E8 (R1) General Considerations for Clinical Studies draft guidelines [19]. Further, the use of master protocol or research networks for specific small disease groups might be effective for promoting clinical trials across multiple regions and countries [20].

In summary, our findings suggest that difficulties exist in coordinating and operating global, multicenter studies that include pediatric populations, possibly due to differences in regulations or administrative procedures across regions. There are several aspects to be considered and addressed, such as developing support systems for clinical trials and improving infrastructure or funding systems [21]

There are three important limitations in this study. First, the approach used here cannot be applied to the clinical trials conducted in countries outside of the EU; therefore, the results and interpretation are not geographically generalizable. Future studies are warranted to investigate the reasons behind the incompletion of pediatric clinical trials in other regions with different advanced regulatory systems, such as the USA and Japan. In Japan, there is no database of clinical trials with study protocols and results presently available. Second, we were unable to take into consideration the details of the study design, such as the duration of intervention and the strength of its invasiveness because of lack of coded data in this database, which would provide a more comprehensive picture of the clinical trials included in the study. Finally, public or academic studies without industrial promotion may suffer more difficulties to complete; however, our study is unlikely to make a concrete conclusion on the impact of public/private study because of the inclusion of a small number of public trials.

## Conclusions

This study is the first study to use EudraCT to clarify the factors associated with failures in the clinical trials that include pediatric patients. Our findings highlight the importance of careful consideration of the study protocol requirements and the feasibility of study logistics, especially for multi-regional clinical trials.

## Supplementary Information

**Additional file 1.**

**Additional file 2.**

**Additional file 3.**

**Additional file 4.**

## Data Availability

The datasets generated and/or analyzed during the current study are available from the [European Medicines Agency How to search the EU Clinical Trials Register] repository, [https://www.clinicaltrialsregister.eu/ctr-search/search].

## References

[1] De Vrueh R, Baekelandt ERF, De Haan JMH Background Paper 6.19:Rare Diseases. 2013. https://www.who.int/medicines/areas/priority_medicines/BP6_19Rare.pdf. Accessed 1 June 2019.

[2] Nakamura H (2015). Efforts toward resolving tasks of off-label or unlicensed use in paediatric patients in Japan [in Japanese]. J Pharm Sci Technol.

[3] Wilson JT (1999). An update on the therapeutic orphan. Pediatrics.

[4] Joseph PD, Craig JC, Caldwell PH (2015). Clinical trials in children. Br J Clin Pharmacol.

[5] Turner MA, Catapano M, Hirschfeld S, Giaquinto C (2014). Paediatric drug development: the impact of evolving regulations. Adv Drug Deliv Rev.

[6] Sekimizu M (2015). PMDAs challenges in pediatric drug development (in Japanese). Regulat Sci Med Products.

[7] Laughon MM, Benjamin DK, Capparelli EV, Kearns GL, Berezny K, Paul IM, Wade K, Barrett J, Smith PB, Cohen-Wolkowiez M (2011). Innovative clinical trial design for pediatric therapeutics. Expert Rev Clin Pharmacol.

[8] Balevic SJ, Cohen-Wolkowiez M (2018). Innovative study designs optimizing clinical pharmacology research in infants and children. J Clin Pharmacol.

[9] Gaasterland CMW, Van der Weide MCJ, Du Prie-Olthof MJ, Donk M, Kaatee MM, Kaczmarek R, Lavery C, Leeson-Beevers K, O'Neill N, Timmis O, Van Nederveen V, Vroom E, Van der Lee JH (2019). The patient's view on rare disease trial design - a qualitative study. Orphanet J Rare Dis.

[10] European Medicines Agency How to search the EU Clinical Trials Register. 1995. https://www.clinicaltrialsregister.eu/doc/How_to_Search_EU_CTR.pdf#zoom=100,0,0. Accessed 2 July 2019.

[11] European Medicines Agency The European Union Clinical Trials Register. 1995. https://www.clinicaltrialsregister.eu/ctr-search/search. Accessed 2 July 2019.

[12] Fogel DB (2018). Factors associated with clinical trials that fail and opportunities for improving the likelihood of success: a review. Contemp Clin Trials Commun.

[13] Kasenda B, von Elm E, You J, Blümle A, Tomonaga Y, Saccilotto R, Amstutz A, Bengough T, Meerpohl JJ, Stegert M, Tikkinen KA, Neumann I, Carrasco-Labra A, Faulhaber M, Mulla SM, Mertz D, Akl EA, Bassler D, Busse JW, Ferreira-González I, Lamontagne F, Nordmann A, Gloy V, Raatz H, Moja L, Rosenthal R, Ebrahim S, Schandelmaier S, Xin S, Vandvik PO, Johnston BC, Walter MA, Burnand B, Schwenkglenks M, Hemkens LG, Bucher HC, Guyatt GH, Briel M (2014). Prevalence, characteristics, and publication of discontinued randomized trials. JAMA.

[14] Dreyer NA, Blackburn S, Hliva V, Mt-Isa S, Richardson J, Jamry-Dziurla A, Bourke A, Johnson R (2015). Balancing the interests of patient data protection and medication safety monitoring in a public-private partnership. JMIR Med Inform.

[15] Nakamura T (2010). Protocol for global clinical trials [in Japanese]. Global Clin Trial.

[16] Kempf L, Goldsmith JC, Temple R (2018). Challenges of developing and conducting clinical trials in rare disorders. Am J Med Genet A.

[17] Giannuzzi V, Altavilla A, Ruggieri L, Ceci A (2016). Clinical trial application in Europe: what will change with the new regulation?. Sci Eng Ethics.

[18] The International Council for Harmonisation ICH E17 guideline. 2018. https://www.pmda.go.jp/int-activities/int-harmony/ich/0022.html?print. Accessed 1 June 2019.

[19] The International Council for Harmonisation ICH E8(R1) guideline. 2019. https://www.ich.org/products/guidelines/efficacy/article/efficacy-guidelines.html#8. Accessed 1 June 2019.

[20] Turner MA, Attar S, De Wildt SN, Vassal G, Mangiarini L, Giaquinto C (2017). Roles of clinical research networks in pediatric drug development. Clin Ther.

[21] Moran C, Smith PB, Cohen-Wolkowiez M, Benjamin DK (2009). Clinical trial design in neonatal pharmacology: effect of center differences, with lessons from the Pediatric Oncology Cooperative Research experience. Clin Pharmacol Ther.

